# Differential Mental Health Impact of COVID-19 Lockdowns on Persons with Non-Communicable Diseases in Trinidad and Tobago

**DOI:** 10.3390/ijerph20166543

**Published:** 2023-08-08

**Authors:** Sandra D. Reid, Shastri Motilal, Shalini Pooransingh, Godfrey St. Bernard, Marsha A. Ivey

**Affiliations:** 1 Psychiatry Unit, Department of Clinical Medical Sciences, Faculty of Medical Sciences, The University of the West Indies (The UWI), Champs Fleurs, Trinidad and Tobago; 2 Public Health and Primary Care Unit, Department of Paraclinical Sciences, Faculty of Medical Sciences, The University of the West Indies (The UWI), Champs Fleurs, Trinidad and Tobago; shastri.motilal@sta.uwi.edu (S.M.); shalini.pooransingh@sta.uwi.edu (S.P.); marsha.ivey@sta.uwi.edu (M.A.I.); 3 Sir Arthur Lewis Institute of Social and Economic Studies, The University of the West Indies (The UWI), St. Augustine, Trinidad and Tobago; godfrey.stbernard@sta.uwi.edu

**Keywords:** COVID-19, non-communicable diseases, mental health, health inequalities, Trinidad and Tobago, marijuana use

## Abstract

Persons with chronic non-communicable diseases (NCDs) were identified as particularly at risk of severe morbidity and mortality during the COVID-19 pandemic. Little is written about the impact of COVID-19 on this sub-population in the Caribbean, where the prevalence of NCDs is disproportionately high. This study aimed to ascertain COVID-related concerns, and the mental health impact of the pandemic among persons with and without NCDs in Trinidad and Tobago, during the acute period of COVID-19 lockdowns early in the pandemic. An anonymous online survey collected cross-sectional data from a convenience sample nationwide. Of 1287 respondents, 219 self-identified as having an NCD. Findings suggest that the pandemic was experienced unequally by persons with NCDs, who were more likely to be concerned about health and wellbeing and to report health inequalities—unemployment, social isolation and negative effects of government restrictions. Compared to those without NCDs, they were more likely to increase use of marijuana during the lockdown period, and to report severe anxiety/depression that can result in exacerbation of NCDs. Interventions for persons with NCDs must address the mental health consequences of any pandemic, including increased drug use, and also address social inequalities to reduce sustained post-pandemic mental health impact and negative health outcomes.

## 1. Introduction

Trinidad and Tobago is a small island developing state located in the Caribbean, with a population of approximately 1.4 million, dominated by two major ethnic groups of African and Indian origin. The population bears a high burden of chronic non-communicable diseases. In 2013 the self-reported prevalence of diabetes was 19.5%, 30.0% for hypertension and 8.2% for heart disease [1]. More than half (55.7%) of the population reported being overweight or obese (BMI > 25); with one-quarter (25.7%) obese [2].

The COVID-19 pandemic significantly impacted the world and adversely affected global and national economies, and the health and well-being of societies. Global communities saw a significant increase in psychological distress and mental health problems. Early studies in China reported that the elderly were at the greatest risk of severe disease. However, the rapidly evolving epidemiological picture shortly identified persons with underlying chronic illnesses as a particularly vulnerable sub-population at high risk for COVID-19 morbidity and mortality [3,4]. Studies from North America, Europe and Africa have recorded higher rates of anxiety and depression among persons with NCDs than those in the general population in response to the pandemic [5,6,7], but there have been no such reports documented in the Caribbean, and little is written on coping behaviours during the lockdown periods of the pandemic. 

Further, COVID-19 has strongly highlighted how social and structural elements in societies can lead to health inequalities and inequities among population groups [8,9]. Indeed, pandemics have been shown to increase the risk of mental disorders due in part to existing inequalities such as poverty and unemployment, as well as the effects of social isolation that accompanied the lockdowns [10]. 

This study aimed to ascertain COVID-related concerns, behavioural coping and mental health impact among persons in Trinidad and Tobago during the acute period of public health lockdowns early in the pandemic. This began from 29 March 2020 with the lockdown of non-essential services, and progressed until 22 June 2020, when beaches, gyms and bars were the last spaces to re-open. This study further examined differences in mental health impact and coping between persons with and without non-communicable diseases. 

## 2. Materials and Methods

The study received ethical approval from the Campus Research Ethics Committee of the University of the West Indies St. Augustine Campus (CREC-SA.0404/06/2020) and the Research Ethics Committee of the Ministry of Health of the Government of Trinidad and Tobago. (# He: 3/13/441 Vol. II).

An online cross-sectional study was conducted of individuals 18 years and older residing in Trinidad and Tobago during the pandemic. Using convenience sampling, participants self-selected for the study via an online self-administered instrument circulated on social media platforms of all the five Regional Health Authorities in Trinidad and Tobago; social media platforms and websites of The University of the West Indies; and through snowball sampling. Acknowledgement of study purpose and provision of online informed consent were required before completion of the survey, after which, a link to the webpage for public mental health services was provided.

The questionnaire, adapted from Australia’s COLLATE study [11], collected information on socio-demographics, self-reported non-communicable chronic disease, religiosity, COVID-19 concerns, pandemic-induced behavioural and mental health changes, the lockdown’s impact on work and social life, financial coping, and perceived positive outcomes. Two validated psychological screening tools were included in the questionnaire: (i) Patient Health Questionnaire 4 (PHQ4) [12] to screen for anxiety and depression and (ii) the positively worded brief measure of hopelessness, Brief-H-Pos [13]. The questionnaire included 45 items and took 10–15 min for completion. 

This paper utilizes the first wave of data collected in the first week of July 2020, after a 3 month lockdown was lifted. The study data were collected and managed using REDCap electronic data capture tools [14]. 

Definitions:

1. Non-communicable disease: Respondents were asked to self-identify with the statement “A person with a chronic illness or pre-existing health condition”.
2. Concerns related to COVID-19—This comprised a list of 27 concerns relating to the COVID-19 pandemic, including fear of infection, impact on work, childcare, domestic violence, overall health and wellbeing, social isolation, public health restrictions, economy, and vaccines. Respondents were asked to select all the concerns they had (Yes/No) and were also able to record other concerns not listed.
3. Behavioural changes—Respondents were asked to record changes in health habits on a 5 point Likert scale—a lot more, a little more, no difference, a little less, and a lot less, compared to before the COVID-19 pandemic. Behavioural health habits included amount of food eaten, amount of exercise, change in sleep pattern, and amount of alcohol, nicotine, and marijuana consumed.
4. Mental health assessment—Core signs and symptoms of anxiety disorder over the prior two weeks were assessed using the ultra-brief four-item Patient Health Questionnaire (PHQ). A score of 3 or greater on either the anxiety or depression sub-scale was accepted as the cut-off point for identifying possible cases of depression or anxiety disorder. PHQ scores range from mild (PHQ score = 3–5) to moderate (PHQ score = 6–8) to severe (PHQ score = 9–12).

### Statistical Analysis

As previously described [15], the data were weighted to align age, sex, and ethnicity with that of the TT population, according to the most recent 2011 census data (Central Statistical Office, Trinidad and Tobago, 2011). All analyses were conducted using the Statistical Package for the Social Sciences v.25 (IBM SPSS Statistics for Windows, Version 25.0: IBM Corp., Armonk, NY, USA), and the significance level was set at 0.05. Respondents who self-identified as having an NCD were compared to those who did not self-identify as having an NCD.

Chi squared analysis and Fisher’s exact test were used to examine associations between the groups in age, sex, and ethnicity, assess differences between health behaviour changes, and PHQ scores.

The frequency of selection for each of the 27 listed concerns about COVID-19 was determined for each group and concerns were ranked. Logistic regression was used to measure associations between selected mental health and behavioural outcomes, and the hypothesized NCD predictor (Yes/No) with adjustment for age and sex. For binomial (Yes/No) outcome variables, each binomial logistic regression model estimated the age- and sex-adjusted Odds Ratio (“adjOR”). In the case of an outcome variable having more than two categories, one of the categories was designated the reference category, and the multinomial logistic regression model estimated adjusted Relative Odds Ratio (“adjROR”).

## 3. Results

Data from 1286 respondents were analysed, of whom 219 self-identified as having an NCD (17.0%). 

### 3.1. Prevalence of NCDs 

The two most prevalent NCDs were hypertension and asthma (Table 1).

Persons with NCDs were more likely to be female and older. There was no difference in prevalence among persons of different ethnicities (Table 2).

### 3.2. Concerns about COVID-19

COVID-19 concerns of persons living with NCDs are ranked in Table 3 from most frequently endorsed (rank = 1) to least frequently endorsed (rank = 28). The most commonly endorsed concern was “Effects on the overall health and wellbeing of family/loved ones”.

### 3.3. Impact of COVID-19 on Persons with and without NCDs

The prevalence of select variables among persons with and without NCDs is shown in Table 4. Persons with NCDs were more likely to report unemployment during the pandemic. Those with NCDs were more likely to self-report very negative effects of government restrictions on mental health compared to those who reported no effects at all. Having an NCD was also associated with a reduced odds of social contact with others outside of family or co-workers (½–2 h compared to none). Such an association did not exist pre-pandemic (Table 4).

### 3.4. COVID-19 Related Changes in Health Behaviour

Persons with NCDS were more likely to sleep more during the COVID-19 lockdown when adjusted for age and sex. No differences were found between persons with NCDs and without NCDs, regarding changes in exercise and eating (Table 5). 

Compared to no change in usage, persons with NCDs who used nicotine had over two times higher odds of less nicotine use during the pandemic. Conversely, respondents with NCDs had over a threefold increased odds of increased marijuana usage compared to no change in use during the pandemic. This was despite similar weekly rates of alcohol, nicotine and marijuana use among the two groups pre-pandemic. These associations occurred independent of age and sex.

### 3.5. Anxiety and Depression

Of all persons with NCDs, 36.4% had a positive anxiety screen vs. 41.9% of respondents without NCDs. (*p* = 0.15) There was no significant difference in the odds of anxiety in those with NCDs (adjOR = 1.15 (0.83–1.59)) after adjustment for age and sex. 

Of the NCD subgroup, less respondents (32.7%) had a positive depression screen compared to those (40%) without NCDs (*p* = 0.047). This significant association was nullified when adjusted for age and sex (adjOR = 1.15 (0.82–1.62)). In respondents with an NCD, the prevalence of those with a severe overall PHQ-4 score (>8) was 25.1% vs. 17.8% in those without NCDs (*p* < 0.001). This association persisted after adjustment for age and sex (adjOR = 2.22 (1.52–3.24)). 

Two hundred and nineteen participants (17%) reported having an NCD, and for this sub-group 61% were males, and the mean age was 45 years (SD 12 years), ranging from 18 to 85 years. Among those with an NCD, the unemployed were at higher odds of severe anxiety/depression compared to those employed (adjOR 4.42), (95% CI: 2.16, 9.04) (Table 6). 

Those reporting being negatively affected by lockdown restrictions were significantly more likely to be severely anxious/depressed compared to those reporting a positive effect or no change (adjOR 2.11) (95% CI: 1.07, 4.18), and those who were socially isolated were more likely to be severely anxious/depressed (adjOR 8.48) (95% CI: 4.06, 17.71).

Among those with an NCD, there were no observed associations of age or sex with severe anxiety/depression. There was also no statistically significant association between having diabetes and severe anxiety/depression.

## 4. Discussion

Trinidad and Tobago, like many countries around the world, is facing a growing burden of noncommunicable diseases. This study reports on the inequitable mental health impact of the COVID-19 pandemic on persons with NCDs compared to those without. Many researchers have recognized the high prevalence of anxiety and depression among patients with NCDs during the pandemic. Ozamiz-Etxebarria [5] reported that among persons with chronic medical illness in Spain, 30.1% reported depression, (16.5% moderate to extremely severe) and 30.7% reported anxiety during COVID lockdowns. In Italy, Mazza et al. (2020) [6] found moderate to severe depression in 32.4% and 18.7% of persons during the pandemic, and this was associated with unemployment, female sex, low educational level, and medical problems. Hajure et al. (2020) [7] noted a prevalence of 55.7% and 61.8% of depression and anxiety, respectively, among chronic medical patients attending hospital follow-up in Ethiopia during the pandemic.

Little epidemiological data exist on mental disorders in Trinidad and Tobago. Prior to the pandemic the estimated lifetime prevalence of mental disorders was 29.9%, with anxiety (17.3%) and mood (11.3%) disorders being the most common. Mental disorders were seen to be more prevalent among females and individuals with a lower educational status and those with chronic medical conditions [16]. 

In this study, the prevalence of depression and anxiety among persons with NCDs at the time of COVID-19 lockdowns was higher than pre-pandemic local reports, and comparable to reports from other countries, with 36.4% of persons who self-reported an NCD screening positive for anxiety, and 32.7% having a positive depression screen. Little baseline data are available for comparison and there are no recent surveys, but the prevalence of depression among patients attending chronic disease clinics in southwest Trinidad was reported as being 28.3% in 2005 [17]. Among small cohorts of persons with diabetes, rates of depression ranged from 26.8% in 2011 [18] to 17.9% in 2013 [19], and 23.4% in 2018 [20]. 

Whether due to pre-existing depression or the impact of the pandemic, or both, it is noteworthy that persons with NCDs were significantly more likely to have scores consistent with more severe anxiety/depression than those without NCDs. Among persons with NCDs, more severe anxiety/depression was associated with social inequalities such as unemployment and social isolation during the COVID-19 lockdown. Unlike previous reports, there was no association between severe depression and age or sex.

A review of the concerns expressed by persons with NCDs about the COVID-19 pandemic suggests that the mental health impact was at least in part related to their medical status. They were significantly more likely to experience concerns about the overall health and wellbeing of themselves and their families; and the availability of food, medicines, and health care, than members of the public who did not have NCDs. The health concerns were likely not specific to the coronavirus since persons with NCDs were no more likely than those without to be concerned about contracting or dying from COVID-19. 

Lockdown measures implemented in the management of the pandemic also have the potential to worsen existing health inequalities among persons with NCDs. In this study, persons with NCDs in Trinidad and Tobago were more likely to be socially isolated during the lockdown, when this was not the experience before the pandemic. Those reporting a negative impact of the lockdown restrictions were significantly more likely to be severely depressed. 

This study also reports an increased use of marijuana among persons with NCDs, to a significantly greater extent than persons without NCDs. While some studies have reported beneficial effects on blood pressure following use of medicinal marijuana [21], others have cautioned that regular marijuana use may increase the risk of kidney disease, heart failure, premature heart attack, and stroke [22,23]. The second most prevalent NCD in this study was asthma. Marijuana has been used medicinally in the management of asthma, and while there may be some benefit of cannabinoids, smoking marijuana can have many harmful effects on the lungs [24]. A rapid review of the effects of recreational marijuana use cautiously concluded that recreational marijuana may increase risk of cardiovascular and renal disease among persons with diabetes [25]. Interestingly, in the current study, among the sub-sample of persons with NCDs, those who self-reported increase in alcohol and marijuana use during the lockdown were less likely to have severe depression. These findings demand further exploration to determine whether increased marijuana use among persons with NCDs was a form of coping with stress, merely recreational, or attempts to self-medicate in the context of restricted health care availability during the pandemic. 

Untreated depression among persons with NCDs is likely to persist beyond the duration of the pandemic and, through a cycle of mutual worsening of both chronic conditions, intensify the morbidity and potential mortality of the medical condition [26]. Further, having an NCD has been established as a major predictor of poor outcomes of COVID-19 [27], and reports are consistent that having COVID-19 worsens underlying NCDs and leads to neglect of care [28]. Without early identification and appropriate intervention, depression has the potential to worsen both the health care outcome of NCDs and overall quality of life [29].

As described by Bambra et al. [30], the lockdown measures may have served to intensify the synergistic effects of social factors (including social isolation), and pandemic effects, contributing to health inequalities among persons with NCDs. This study suggests that, in Trinidad and Tobago, the COVID-19 pandemic was experienced unequally by persons with NCDs, resulting in more severe mental health effects that can exacerbate NCDs. The findings further begin to fill an existing gap of knowledge by documenting some of the associated social and structural risk factors associated with the high rates of serious anxiety and depression in the population of persons with NCDs in a small island developing state in the Caribbean. There is a real threat of further global pandemics. In addressing the medical care of persons with NCDs, public health responses and health policies in Caribbean states must not fail to consider their mental health, the social inequalities that predispose them to poor mental health and the type of coping employed. When addressed, these are likely to have significant positive impact on the outcome of both the mental health challenge and the underlying chronic medical illness.

This study is not without limitations. NCDs were self-reported and not clinically verified. The data collection method was limited by prevailing COVID-19 public health restrictions and the sampling strategy would not result in a representative sample, thus limiting generalization of the results. The authors aimed to minimize this by using weighted data. In addition, those persons with NCDs who were worst affected by depression might have been least likely to respond to the survey. Lastly, multiple comparisons were made in the analyses, which could have inflated the Type 1 error rate, potentially leading to false positive findings.

## 5. Conclusions

Notwithstanding the limitations, the findings of this study clearly identify the vulnerability of the population of persons with NCDs who are disproportionately affected by the possible interactions of the chronic medical condition, underlying social inequalities, and increased susceptibility to pandemic effects. Interventions for persons with NCDs must address the mental health consequences of any pandemic, especially among vulnerable groups where syndemic interactions may occur; and any mental health intervention should address social inequalities to reduce sustained post-pandemic mental health impact and negative health outcomes.

## Figures and Tables

**Table 1:** Prevalence of self-reported chronic non-communicable diseases among study subjects.

Chronic Disease (N *)	Yes	%
Hypertension (N = 1221)	193	15.8
Asthma (N = 1182)	175	14.8
Diabetes (N = 1202)	92	7.7
Cardiac disease (N = 1158)	43	3.7
Cancer (N = 1135)	38	3.2
Chronic Obstructive Pulmonary Disease/emphysema (N = 1149)	9	0.8
HIV (N = 1146)	6	0.5

* Missing values excluded.

**Table 2:** Age, sex, and ethnicity of study subjects with and without chronic non-communicable diseases.

Demographic	Persons with NCDs		*p*-Value
	No (*n* = 1067)	Yes (*n* = 219)	
Age (years)			
18–24	255 (23.9%)	16 (7.3%)	**<0.001 ^†^**
25–39	344 (32.2%)	51 (23.2%)	**0.008 ^†^**
40–54	302 (28.3%)	92 (41.8%)	**<0.001 ^†^**
55–75	161 (15.1%)	60 (27.3%)	**<0.001 ^†^**
>76	5 (0.5%)	1 (0.5%)	0.999 *
Sex			
Male	547 (51.3%)	85 (38.6%)	**<0.001 ^†^**
Female	514 (48.2%)	132 (60.0%)	**0.001 ^†^**
Other	6 (0.6%)	3 (1.4%)	0.188
Ethnicity			0.758 *
African	397 (37.2%)	84 (38.4%)	
East Indian	406 (38.1%)	71 (32.4%)	
Other	264 (24.7%)	64 (29.2%)	

* Chi square test, ^†^ Fishers exact test, Bolded values indicate *p* < 0.05 (two-sided).

**Table 3:** COVID-19 concerns of study subjects living with a chronic non-communicable disease.

Ranked Concerns of Persons with NCDs (N = 219)	Yes	%	* adjOR (95% CI)	*p* Value
Effects on the overall health and wellbeing of family/loved ones	132	60.3	**1.54 (1.14–2.09)**	**0.005**
2. Trinidad and Tobago economy	132	60.3	0.81 (0.59–1.10)	0.179
3. Catching COVID-19	128	58.4	1.049 (0.77–1.42)	0.760
4. Effects on my overall health and wellbeing	128	58.4	**1.59 (1.17–2.15)**	0.003
5. A loved one catching COVID-19	121	55.3	0.82 (0.61–1.11)	0.195
6. Rising cost of living	116	53.0	**0.85 (0.63–1.15)**	**0.293**
7. Effects on the overall health and wellbeing of society	115	52.5	1.11 (0.83–1.51)	0.468
8. Uncertainty about the future	109	49.8	0.91 (0.67–1.22)	0.516
9. Personal finances	108	49.3	1.05 (0.78–1.42)	0.750
10. A loved one dying from COVID-19	106	48.4	0.85 (0.63–1.14)	0.270
11. World economy	96	43.8	0.75 (0.55–1.01)	0.057
12. Availability of food and medicines	91	41.6	**1.81 (1.33–2.46)**	**<0.001**
13. Availability of a vaccine for COVID-19	81	37.0	1.06 (0.77–1.46)	0.714
14. Side effects of a vaccine for COVID-19	78	35.6	1.14 (0.82–1.56)	0.430
15. Access to appropriate medical care	77	35.2	**1.55 (1.13–2.15)**	**0.008**
16. Dying of COVID-19	75	34.2	1.01 (0.73–1.38)	0.962
17. Need to wear masks	75	34.2	**1.48 (1.08–2.05)**	**0.017**
18. Risk of unemployment or reduced employment	74	33.8	**0.78 (0.56–1.06)**	**0.114**
19. Travel restrictions	69	31.5	1.10 (0.80–1.03)	0.559
20. Social isolation	56	25.6	1.15 (0.81–1.64)	0.433
21. Need for continued physical distancing	50	22.8	0.89 (0.63–1.27)	0.533
22. Fear of those who had/were exposed to COVID-19	48	21.9	0.92 (0.64–1.33)	0.659
23. Not being able to attend regular place of worship	47	21.5	**1.25 (0.85–1.82)**	**0.252**
24. Balancing work and caring for children/dependents	37	16.9	0.74 (0.50–1.09)	0.131
25. Adapting to working from home (e.g., IT/connectivity issues	33	15.1	1.34 (0.87–2.06)	0.186
26. Media coverage of the pandemic	15	6.8	0.47 (0.27–0.83)	0.009
27. Others (please specify)	4	1.8	0.37 (0.12–1.15)	0.085
28. Domestic violence	1	0.5	0.25 (0.03–1.91)	0.181

* adjOR—Age- and sex-adjusted odds ratio for outcome in those with NCD; Bolded values indicate *p* < 0.05 (two-sided) for the null hypothesis that adjOR = 1.

**Table 4:** Prevalence of select variables among study subjects with and without an NCD.

Select Variables	Prevalence among Those		* adjROR (95% CI)	*p* Value
	With No NCDs	With NCDs		
**Employment Status Before Pandemic**				
Employed full time/part time	664 (63.0%)	147 (68.1%)	1	
Student full time/part time	211 (20.0%)	14 (6.5%)	0.65 (0.28–1.47)	0.299
Unemployed	64 (6.1%)	19 (8.8%)	1.61 (0.93–2.81)	0.091
Homemaker/volunteer/retired	115 (10.9%)	36 (16.7%)	0.80 (0.48–1.34)	0.393
**Current Employment Status**				
Employed full time/part time	601 (56.9%)	117 (53.7%)	1	
Student full time/part time	179 (17.0%)	12 (5.5%)	0.68 (0.29–1.60)	0.381
Unemployed	167 (15.8%)	44 (20.2%)	**1.49 (1.01–2.22)**	0.047
Homemaker/volunteer/retired	109 (10.3%)	45 (20.3%)	1.41 (0.86–2.30)	0.171
**Job Loss Due to Pandemic**				
No	86 (52.4%)	22 (50.0%)	1	
No but reduced hours	21 (12.8%)	2 (4.5%)	0.20 (0.04–1.03)	0.054
No but job loss expected	12 (7.3%)	1 (2.3%)	0.26 (0.03–2.32)	0.226
Yes	45 (27.4%)	19 (43.2%)	1.34 (0.63–2.86)	0.442
**Social Contact with Others (Not Family or Co-Workers)** **In a typical week, before the COVID-19 pandemic, how much time would you have spent with people who do not live with you (not related to work)?**				
No time	69 (6.6%)	21 (9.6%)	1	
Less than ½ h daily	165 (15.7%)	37 (17.0%)	0.71 (0.38–1.32)	0.281
½ h–1 h daily	196 (18.6%)	46 (21.1%)	0.74 (0.40–1.36)	0.329
1 h–2 h daily	160 (15.2%)	38 (17.4%)	0.66 (0.35–1.23)	0.191
More than 2 h daily	463 (44.0%)	76 (34.9%)	0.64 (0.36–1.13)	0.125
**Social Contact with Others (Not Family or Co-Workers)** **In the last week, how much time have you spent each day in contact with people who do not live with you (not related to work)?**				
No time	152 (14.4%)	51 (23.4%)	1	
Less than ½ h daily	259 (24.5%)	48 (22.0%)	**0.51 (0.33–0.81)**	0.004
½ h–1 h daily	282 (26.7%)	51 (23.4%)	**0.55 (0.35–0.86)**	0.009
1 h–2 h daily	199 (18.8%)	33 (15.1%)	**0.49 (0.30–0.81)**	0.005
More than 2 h daily	164 (15.5%)	35 (16.1%)	0.63 (0.38–1.04)	0.069
**Self-Reported Negative Mental Effects of Government Restrictions**				
Not at all	454 (43.2%)	94 (43.3%)	1	
Very positively	81 (7.7%)	11 (5.1%)	0.56 (0.29–1.11)	0.099
Somewhat positively	175 (16.7%)	27 (12.4%)	0.86 (0.54–1.39)	0.547
Somewhat negatively	304 (29.0%)	68 (31.3%)	1.40 (0.98–2.02)	0.066
Very negatively	36 (3.4%)	17 (7.8%)	**2.97 (1.55–5.66)**	0.001

* Adjusted for age and sex; adjROR: category-specific adjusted odds ratio for outcome in those with NCDs divided by reference category’s adjusted odds ratio; Bolded values indicate *p* < 0.05 (two-sided) for the null hypothesis that adjROR = 1.

**Table 5:** Changes in health behaviour during COVID-19 lockdown among study subjects with and without NCDs.

Health Behaviours	Persons with an NCD		* adjROR (95% CI)	*p* Value
	No (*n* = 1067)	Yes (*n* = 219)		
**Amount you Eat Daily**				
No change	184 (17.5%)	50 (23.4%)	1	
More	607 (57.0%)	98 (45.8%)	0.72 (0.49–1.06)	0.097
Less	260 (24.7%)	66 (30.8%)	1.30 (0.84–2.01)	0.232
**Exercise Daily**				
No change	298 (28.2%)	57 (26.8%)	1	
More	290 (27.5%)	48 (22.5%)	1.03 (0.67–1.59)	0.881
Less	467 (44.3%)	108 (50.7%)	1.38 (0.96–1.99)	0.079
**Sleep Daily**				
No change	284 (26.8%)	52 (23.7%)	1	
More	461 (43.6%)	100 (45.7%)	**1.80 (1.21–2.67)**	0.003
Less	313 (29.6%)	67 (30.6%)	1.41 (0.94–2.12)	0.102
**Weekly Alcoholic Drink BEFORE Lockdown**				
I do not drink	503 (47.6%)	113 (51.6%)	1	
<10 standard drinks /week	521 (49.3%)	102 (46.6%)	0.88 (0.65–1.19)	0.401
>10 standard drinks/week	32 (3.0%)	4 (1.8%)	0.48 (0.15–1.49)	0.202
**Alcohol Drinking Change**				
No change	597 (63.2%)	111 (56.1%)	1	
More	139 (14.7%)	33 (16.7%)	1.52 (0.98–2.38)	0.064
Less	208 (22.0%)	54 (27.3%)	1.40 (0.96–2.05)	0.078
**Nicotine Use BEFORE Lockdown**				
I do not use nicotine	970 (92.4%)	193 (91.0%)	1	
<3 times/week	13 (1.2%)	2 (0.9%)	0.88 (0.18–4.24)	0.874
>3 times/week	67 (6.4%)	17 (8.0%)	1.13 (0.62–2.04)	0.694
**Nicotine Use Change DURING Lockdown**				
No change	702 (91.1%)	136 (92.5%)	1	
More	49 (6.4%)	4 (2.7%)	0.48 (0.20–1.18)	0.109
Less	20 (2.6%)	7 (4.8%)	**2.48 (1.01–6.06)**	0.047
**Marijuana use BEFORE lockdown**				
I do not use marijuana	910 (86.1%)	205 (94.9%)	1	
<3 times/week	101 (9.6%)	7 (3.2%)	0.46 (0.20–1.10)	0.082
>3 times/week	46 (4.4%)	4 (1.9%)	0.66 (0.23–1.91)	0.443
**Marijuana frequency change DURING lockdown**				
No change	676 (87.2%)	128 (87.1%)	1	
More	43 (5.5%)	16 (10.9%)	**3.31 (1.81–6.06)**	<0.001
Less	56 (7.2%)	3 (2.0%)	0.97 (0.41–2.33)	0.953

* Adjusted for age and sex; adjROR: category-specific adjusted odds ratio for outcome in those with NCDs divided by reference category’s adjusted odds ratio; Bolded values indicate *p* < 0.05 (two-sided) for the null hypothesis that adjROR = 1.

**Table 6:** Associations between severe PHQ-4 scores and selected behavioural/social factors among study subjects with self-reported NCDs.

Factors	PHQ-4 Anxiety/Depression Score *				adjROR ^Ϯ^	95% CI	*p* Value
	Not Severe		Severe				
	*n*	%	*n*	%			
Employment Status before Pandemic							
Employed	118	74.7	26	48.1	1		
Unemployed/Student/ Homemaker/Retired	40	25.3	28	51.9	**4.42**	**(2.16, 9.04)**	**<0.001**
Job Loss due to Pandemic							
No change	6	33.3	19	73.1	1		
Lost job	12	66.7	7	29.9	**0.19**	**(0.05, 0.76)**	**0.019**
Social Isolation							
No	139	85.8	23	41.8	1		
Yes	23	14.2	32	58.2	**8.48**	**(4.06, 17.71)**	**<0.001**
Self-Reported Change in Mental Effects from Government Restrictions							
No Change	88	55	6	11.1	1		
Positive	20	12.5	16	29.6	**12.04**	**(4.03, 35.96)**	**<0.001**
Negative	52	32.5	32	59.3	**6.35**	**(2.39, 16.89)**	**<0.001**
Self-Reported Change in Mental Effects from Government Restrictions							
No Change/Positive	108	67.5	22	40.7	1		
Negative	52	32.5	32	59.3	**2.11**	**(1.07, 4.18)**	**0.032**
Alcohol drinking change							
No Change	75	52.1	35	68.6	1		
More	27	18.8	5	9.8	**0.21**	**(0.07, 0.65)**	**0.007**
Less	42	29.2	11	21.6	**0.39**	**(0.16, 0.95)**	**0.038**
Alcohol drinking change							
No Change/Less	117	81.3	45	90.0	1		
More	27	18.8	5	10.0	**0.30**	**(0.11, 0.88)**	**0.028**
Marijuana Use Change Lockdown							
No Change	85	87.6	40	90.9	1		
More	12	12.4	4	9.1	**0.19**	**(0.04, 0.79)**	**0.022**

* Based on Patient Health Questionnaire [12] (PHQ > 8) for severe cases ^Ϯ^ adjROR: category-specific age- and sex-adjusted odds ratio for outcome in those with severe anxiety/depression divided by reference category’s adjusted odds ratio. Bolded values indicate *p* < 0.05 (two-sided) for the null hypothesis that adjROR = 1.

## Data Availability

The data is not publicly accessible but can be requested from the corresponding author.
